# Correction: DFT design of PO_4_@C_60_ nanohybrid unveils superelectrophilic molecular metamorphosis with molecular dynamics exhibiting therapeutic potential against p53 cancer mutant Y220C

**DOI:** 10.1039/d6ra90119k

**Published:** 2026-09-16

**Authors:** Ryan John Laverne, Sudheesh K. Shukla, Ashok Kumar Mishra

**Affiliations:** a Department of Physics, Dr Shakuntala Misra National Rehabilitation University Lucknow Uttar Pradesh–226017 India akmishra@dsmnru.ac.in akmishra2k5@gmail.com; b Department of Biosciences, Graphic Era (Deemed to be University) Dehradun Uttarakhand–248002 India; c Department of Chemical Sciences, University of Johannesburg, Doornfontein Campus P.O. Box 17011 Johannesburg 2028 South Africa sudheeshs@uj.ac.za

## Abstract

Correction for ‘DFT design of PO_4_@C_60_ nanohybrid unveils superelectrophilic molecular metamorphosis with molecular dynamics exhibiting therapeutic potential against p53 cancer mutant Y220C’ by Ryan John Laverne *et al.*, *RSC Adv.*, 2026, **16**, 32744–32762, https://doi.org/10.1039/d6ra01727d.

The authors regret that one of the authors (Sudheesh K. Shukla) was not designated as a corresponding author in the original article. The corrected author list is as shown above.

The Royal Society of Chemistry apologises for these errors and any consequent inconvenience to authors and readers.

